# A Potential Role of Adropin in Inflammatory Rheumatic Diseases—What Do We Know So Far?

**DOI:** 10.3390/biomedicines13092300

**Published:** 2025-09-19

**Authors:** Petra Simac, Marin Petric, Marijana Jankovic Danolic, Dijana Perković

**Affiliations:** 1 Division of Rheumatology and Clinical Immunology, Department of Internal Medicine, University Hospital of Split, 21000 Split, Croatia; petra_simac@hotmail.com (P.S.); marin.petric2017@gmail.com (M.P.); 2 School of Medicine, University of Split, 21000 Split, Croatia; marijana-st@hotmail.com

**Keywords:** adropin, autoimmune diseases, biomarker, cardiovascular diseases, endothelial dysfunction, metabolic homeostasis, oxidative stress

## Abstract

Adropin is a regulatory peptide hormone involved in metabolic homeostasis, cardiovascular protection, and immune modulation. Recent evidence suggests that adropin plays a role in the pathophysiology of autoimmune rheumatic diseases (ARDs) by influencing key processes such as endothelial function, oxidative stress, tissue fibrosis, and immune cell regulation. This review summarizes current knowledge on adropin’s biological functions and its relevance in conditions including rheumatoid arthritis, systemic lupus erythematosus, systemic sclerosis, primary Sjögren’s syndrome, osteoarthritis, psoriasis, Behçet’s disease, and Kawasaki disease. We discuss how adropin interacts with various signaling pathways and highlight its potential role in macrophage polarization, regulatory T cell activity, and fibrotic remodeling. Although data remain limited and sometimes conflicting, altered adropin levels have been observed across several ARDs, suggesting potential utility as a biomarker or therapeutic target. Further research is needed to clarify its clinical significance and translational potential in immune-mediated diseases.

## 1. Introduction

**Figure 1:**
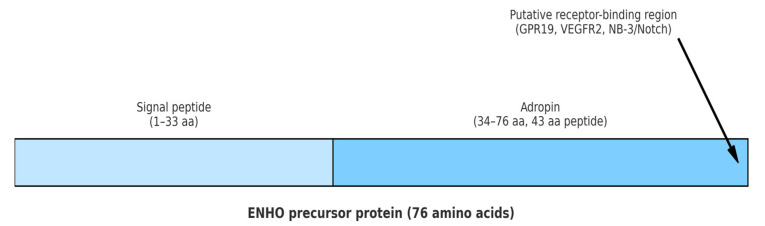
Structural schematic of adropin peptide. The *Enho* precursor protein consists of 76 amino acids. The N-terminal region (1–33 aa) forms the signal peptide, while the C-terminal region (34–76 aa) represents the functional adropin peptide (43 aa). The C-terminal part contains the putative receptorbinding region, implicated in interactions with GPR19, VEGFR2, and NB-3/Notch. Abbreviations: *Enho*: energy homeostasis-associated gene; aa: amino acids; GPR19: G-protein coupled receptor 19; VEGFR2: vascular endothelial growth factor receptor 2; NB-3/Notch: neuroblastoma suppressor of tumorigenicity 1/Notch signaling pathway.

Adropin is a regulatory peptide discovered in 2008 by Kumar et al., consisting of 43 amino acids. It is produced by proteolytic cleavage of a 76 amino acid precursor. Adropin is encoded by the energy homeostasis-associated (*Enho*) gene and secreted primarily by liver and brain, but it could also be found in peripheral mononuclear blood cells, breast cancer cells, small intestine, endothelial cells (ECs), heart, muscles and kidneys [1,2,3]. The gene-encoding *Enho* is located on chromosome 9p13.3 and consists of 25 exons [2]. *Enho* messenger ribonucleic acid (mRNA) expression in the liver is affected by the amount of carbohydrates and fats in the diet and is regulated by a liver X receptor alpha (LXRα), which is involved in cholesterol and triglyceride metabolism. Reduced *Enho* mRNA expression has been demonstrated in obese mice fed with hypercaloric diet for 2 months, while exposure to a high-fat diet in a shorter period (up to 1 month) results in enhancement of *Enho* mRNA expression [2,3]. Consequently, it is assumed that adropin plays a pivotal role in glucose homeostasis and lipid metabolism leading to a reduction in obesity and insulin resistance (IR) [1,4]. Similarly to the *Enho* mRNA expression, serum adropin levels in humans are affected by diet composition, as well as by the body weight [5]. There is a positive correlation of serum adropin concentration with fat intake, and a negative association with carbohydrate intake and body mass index (BMI) [6,7,8]. Women have lower serum adropin levels than men [6,9]. Even though cholesterol suppresses the expression of *Enho* mRNA and reduces adropin levels in humans, which is in concordance with the results of in vivo and in vitro studies, a direct involvement of adropin in cholesterol metabolism has not been established yet [9,10]. Interestingly, there is a negative correlation of adropin with low-density lipoprotein cholesterol levels in men [6,9]. Adropin levels in human plasma range from 1 to 10 μg/L and tend to decrease with age [9]. Adropin also acts as a secretory peptide through three distinct extracellular membrane receptors: orphan G-protein coupled receptor-19 (in cardiac cells, central nervous system and in metastatic breast cancer); Nb-3/Notch signaling pathway (in mice brains) and vascular endothelial growth factor receptor 2—VEGFR2 (in endothelium) [11,12,13,14]. The structure of adropin is shown in Figure 1.

Although more human studies are required to confirm functional role of adropin in controlling adiposity, lipid and glucose metabolism, cardiovascular system and inflammation, awareness of its importance is rising [5]. In this review, we aim to summarize current evidence on serum adropin levels in systemic autoimmune rheumatic diseases (ARDs) and to examine the potential role of adropin in modulating the complex pathophysiological pathways involved in these disorders.

## 2. Methods

This narrative review was conducted to synthesize current evidence on the role of adropin in ARDs. A comprehensive literature search was performed in three major biomedical databases: PubMed, Scopus, and Web of Science, covering studies published up to 31 May 2025. The search strategy included combinations of relevant keywords and MeSH terms, such as “adropin”, “autoimmune rheumatic diseases”, “endothelial dysfunction”, “metabolic homeostasis”, “oxidative stress”, “psoriasis”, “cardiovascular diseases”, “inflammation” and “biomarkers”. Eligible sources included original research articles, experimental studies, and relevant review articles published in English. Studies were selected based on their relevance to the review objective, with particular focus on the expression, regulation, physiological roles, and clinical implications of adropin in the context of systemic inflammation and ARDs. As this is a narrative review, no systematic review protocol was followed, and no formal quality assessment of the included studies was conducted. The reference lists of retrieved articles were also screened manually to identify the additional relevant literature. It is important to note that a systematic review was not performed due to the heterogeneity of the included studies, limited availability of quantitative data, and the exploratory nature of the topic.

## 3. Mechanisms of Adropin Action

### 3.1. Metabolic Mechanisms

Extensive research has primarily focused on the role of adropin in obesity, glucose and lipid metabolism, cardiovascular diseases, liver disease, reproductive health, and cancer, with particular emphasis on its role in energy homeostasis due to its multiple actions in improving metabolic disorders [5,10].

The key regulator of energy balance is adipose tissue, which acts as a junction of energy homeostasis, inflammation, and atherosclerosis. When the storage capacity of adipocytes for free fatty acids is exceeded, inflammatory signaling pathways are activated, leading to adipocyte dysfunction, IR, and the development of type 2 diabetes. Activated pro-inflammatory macrophages within adipose tissue are key mediators in this process, primarily through secretion of tumor necrosis factor α (TNF-α) [15]. Adropin regulates lipogenesis by modulating expression of peroxisome proliferator activated receptor-γ (PPAR-γ) and through activation extracellular signal-regulated kinase 1/2 (ERK1/2) and protein kinase B (AKT) signaling pathways [1,10]. PPAR-γ, a nuclear transcription factor with an irreplaceable role in fat cell metabolism, regulates adipocyte differentiation and fatty acid transport, and affects adipokine secretion (adiponectin, resistin), pro-inflammatory cytokines (TNF-α, IL-interleukin-6), and monocyte chemoattractant protein-1 [16]. By promoting PPAR-γ activity and inhibiting preadipocyte differentiation via ERK1/2 and AKT, adropin reduces the pro-inflammatory activity of macrophages and demonstrates anti-inflammatory effects [5,17,18]. An imbalance in immune cell populations contributes to adipose tissue inflammation and IR [19]. Animal studies showed that adropin administration suppressed lipogenic gene expression, improved insulin sensitivity, and reduced fasting insulin and triglyceride levels. In conclusion, excessive secretion of adropin positively correlates with improved glucose tolerance, reduced IR, and accelerated carbohydrate oxidation [1,3]. Adropin increases hepatic insulin sensitivity and suppresses glucose production in hepatocytes by increasing of insulin receptor substrate-1, -2, and AKT phosphorylation and interfering with cyclic adenosine monophosphate/protein kinase A signaling pathway [1,3]. Similar effects of adropin on glucose metabolism in striated muscle have also been reported, including reduced glucose utilization and increased pyruvate dehydrogenase activity, which promotes glycolysis [3].

### 3.2. Immune Mechanisms

Previous studies have demonstrated multiple anti-inflammatory effects of adropin on immune cells and cytokines, confirming its important immunomodulatory role [18]. Low adropin levels alter immune cell homeostasis and cytokine profiles, promoting sustained inflammation. The involvement of adropin has been investigated in pathophysiology of the numerous chronic inflammatory conditions, including autoimmune diseases, although the specific underlying mechanisms have not been fully elucidated [3,18,20,21,22,23,24,25].

One of the key mechanisms through which adropin exerts its anti-inflammatory effect is the regulation of macrophage polarization. M1 macrophages secrete pro-inflammatory cytokines, whereas M2 macrophages exhibit anti-inflammatory and tissue-regenerative properties [26,27,28]. Adropin promotes polarization toward the anti-inflammatory M2 phenotype [29].

Another important pathway involves regulatory T cells (Tregs). Evidence suggests that adropin deficiency may impair Tregs function or reduce their numbers, thereby contributing to the onset of autoimmune disease [16,18,30]. Tregs regulate T helper lymphocyte activity, which plays a central role in orchestrating immune responses [19]. Similarly to macrophages, Tregs play a crucial role in controlling inflammation within adipose tissue [31]. In obesity, macrophage infiltration into adipose tissue promotes the release of TNF-α and monocyte chemoattractant protein-1, resulting in Tregs depletion and the accumulation of macrophages. In that way, a chronic inflammatory state is perpetuated [5]. Several studies have investigated the regulatory effects of adropin on Tregs through distinct signaling pathways [16,32]. For instance, in mice fed a high-fat diet, adropin deficiency worsened Tregs depletion and contributed to the development of fatty pancreas and type 2 diabetes [30].

Animal models also show that adropin deficiency increases pro-inflammatory gene expression. Adropin knockout mice exhibit upregulation of IL-6, IL-1β, and TNF-α [14,33]. Retinoid-related orphan receptor α-deficient staggerer mice, when fed a high-fat diet, also showed increased mRNA expression of these cytokines [34,35]. These mice normally downregulate cytokine-mediated responses by inhibiting nuclear factor kappa B (NF-κB) signaling [36]. NF-κB, a master regulator of chemokine/cytokine transcription, can alter *Enho* expression and thereby serum adropin levels [37]. Similar findings have been reported in non-alcoholic steatohepatitis models and diabetic rats [30,33].

### 3.3. Vascular Mechanisms

Adropin has protective effects on the cardiovascular system, including the heart and vasculature [2]. It improves angiogenesis, blood flow, and capillary density, and protects ECs, thereby enhancing cardiac and coronary function. Mechanistically, adropin promotes endothelial nitric oxide synthase (eNOS) activity via VEGFR2–ERK1/2–AKT signaling, increasing nitric oxide (NO) bioavailability [14]. NO is essential for endothelial homeostasis and prevents leukocyte and monocyte adhesion [38]. Through this pathway, adropin reduces endothelial inflammation and TNF-α–mediated leukocyte extravasation [39]. In vitro, adropin prevents THP-1 monocyte adhesion to ECs and inhibits NF-κB activation, reducing cytokine-driven atherosclerosis [18,30]. Adropin also regulates inducible nitric oxide synthase expression, lowering TNF-α and IL-6 [13]. Conversely, adropin deficiency leads to upregulation of inflammatory genes (IL-1β, IL-6, TNF-α), contributing to vascular inflammation, lipotoxicity, IR, and oxidative stress [5].

### 3.4. Adropin’s Interactions with Molecular and Inflammatory Pathways in ARDs

As previously mentioned, adropin intersects with two central transcriptional regulators of inflammation: NF-κB and PPAR-γ. Current evidence indicates that adropin deficiency disrupts NF-κB–dependent transcription, leading to increased expression of IL-1β, IL-6, and TNF-α [33,34,35,36]. Since these are the main pro-inflammatory cytokines reported in ARDs where adropin has been studied, it may be concluded that adropin regulation in ARDs depends on several factors, and that the pro-inflammatory milieu itself can alter circulating adropin levels. In addition, NF-κB has been shown to regulate *Enho* gene expression, suggesting a bidirectional relationship between adropin and inflammatory signaling [37]. This link is supported by observations in synovial inflammation in rheumatoid arthritis (RA) and osteoarthritis (OA), vascular activation in collagenoses, and metabolic inflammation in obesity, all conditions in which NF-κB acts as a key driver [37,40,41,42].

PPAR-γ is a nuclear receptor that regulates lipid metabolism and is closely connected with adropin, particularly in relation to macrophage polarization, where both promote an anti-inflammatory phenotype [29]. Impairment of PPAR-γ has been reported in systemic sclerosis (SSc), where antifibrotic responses are reduced. Notably, recombinant adropin treatment reduced collagen deposition in SSc skin, consistent with a PPAR-γ–mediated antifibrotic effect [43]. Taken together, these data position adropin as a modulator that hinder NF-κB activation while enhancing PPAR-γ dependent anti-inflammatory and antifibrotic pathways across multiple ARDs.

Beyond transcriptional control, adropin may regulate innate and adaptive immunity through the NLR family pyrin domain–containing 3 (NLRP3) inflammasome. In metabolic and cardiovascular contexts, adropin has been shown to suppress NLRP3 activity by reducing reactive oxygen species and IL-1β release and by attenuating AKT/GSK3β/NF-κB/NLRP3 signaling. These findings suggest that adropin may act as a context- and dose- dependent modulator of inflammasome-driven inflammation, a hypothesis that should be studied in ARDs [32,44]. In high-fat-diet models, adropin deficiency aggravated inflammasome priming, leading to excess IL-1β production and systemic metabolic inflammation [30]. Given the central role of IL-1β in arthritis and vasculitides, these findings suggest that adropin may serve as a negative regulator of NLRP3 activation in ARDs. Adropin also appears to intersect indirectly with the janus kinase–signal transducer and activator of transcription (JAK–STAT) axis, which is central to cytokine-driven autoimmunity. While direct binding to STAT proteins has not been demonstrated, adropin reduces upstream cytokine secretion, notably TNF-α and IL-6, thereby con-straining STAT-dependent transcriptional activation. In RA synoviocytes, where IL-6/JAK–STAT signaling drives synovitis, and in systemic lupus erythematosus (SLE), where interferon signatures dominate, adropin deficiency could contribute to unchecked STAT signaling [32]. These observations highlight the need to further investigate adropin as a modulator of inflammasome and JAK–STAT activity in human ARDs.

## 4. Clinical Evidence in Autoimmune Rheumatic Diseases

The levels of circulating adropin were investigated in disorders with low-grade chronic inflammation, such as type 2 diabetes, atherosclerosis, coronary artery disease, arterial hypertension, obstructive sleep apnea, and in patients on hemodialysis [8,32,39,45,46,47,48]. These data indicate that adropin-mediated immunological and inflammatory processes are involved in various pathological conditions.

Adropin deficiency and/or *Enho* mutations play a key role in lung damage caused by myeloperoxidase anti-neutrophil cytoplasmic antibodies (MPO-ANCA). The underlying pathophysiological mechanism involves activation of ECs during leukocyte migration, mediated by pro-inflammatory cytokines (IL-1 and TNF-α). In vitro studies in human ECs demonstrated that adropin deficiency reduced AKT1 and eNOS phosphorylation and increased vascular cell adhesion molecule-1 expression, while peripheral blood mononuclear cells from patients with MPO-ANCA vasculitis carrying *Enho* mutations showed reduced circulating adropin. In vivo, adropin knockout mice developed pulmonary vasculitis with increased pro-inflammatory cytokines and loss of Treg cells, closely resembling human MPO-ANCA associated lung injury [49].

Interestingly, serum adropin levels in psoriatic patients with metabolic syndrome were significantly lower compared to those without metabolic syndrome, although all patients suffering from psoriasis have lower serum adropin levels compared to the healthy controls. Low adropin levels increase the risk of developing metabolic syndrome in individuals with psoriasis [50].

So far, serum adropin levels have been investigated in several ARDs [21,22,23,24,25,43,51]. The results of published studies suggest that adropin have a role in the complex pathophysiology of these diseases. Among patients with OA and RA, decreased serum adropin concentrations were found when compared to healthy controls, except in one small cohort of RA patients who did not have reduced adropin levels, but had reduced *Enho* gene expression [22,23,25]. In OA, serum adropin levels are negatively correlated with both the severity of knee OA and inflammatory markers, including TNF-α, white blood cell count, and neutrophil-to-lymphocyte ratio [23]. There are several possible explanations for these correlations. One of the proposed explanations is the interaction of adropin with eNOS and pro-inflammatory cytokines, especially TNF-α, IL-6, and IL-1β [39]. Adropin, by altering the activity of eNOS through PI3K-Akt and ERK1/2 signaling pathways and activating VEGFR2, reduces TNF-α and IL-6 induced oxidative stress in macrophages and ECs. Furthermore, adropin interferes with TNF-α-mediated leukocyte extravasation, thereby creating an anti-inflammatory environment [14]. It is important to note that the inflammatory milieu of synovial fluid, composed of TNF-α and IL-1β, plays a key role in the onset and progression of knee OA [52]. Additionally, serum levels of adropin and TNF-α are inversely correlated, which further supports their involvement in inflammation. Another important result of this study is significant lower serum adropin levels in knee OA patients with BMI > 30 [23]. In our study, a statistically significant negative correlation was observed between serum adropin levels and parameters of glucose metabolism [25]. These findings support a complex relationship among adropin, metabolism and inflammation. It is also important to mention the NF-κB, a key regulator of synovial inflammation in both OA and RA. In both diseases, its activity is dysregulated, leading to an imbalance of chemokines, cytokines, and adhesion molecules. This imbalance may be associated with serum adropin levels as well as *Enho* expression [25,37,41,53]. Based on these findings, it can be concluded that adropin interferes with inflammation in both OA and RA [23,25].

To date, only one other study in patients with RA and SLE has reported increased *Enho* expression in RA subjects although no differences in serum adropin levels were observed between the study groups which is not consistent with the findings of our study, where RA patients had low serum adropin levels. This could be explained by smaller sample size in their study, notable heterogeneity between study groups, variations in exclusion criteria, a younger participant population, and a markedly shorter RA duration. Some of these differences are important, especially the duration of RA and the age of the patient, as they may influence serum adropin levels [22,25]. Interestingly, in both studies no association was found between serum adropin levels and disease activity indices. Specifically, in our study adropin did not correlate with the Disease Activity Score-28-erythrocyte sedimentation rate or with the Health Assessment Questionnaire, while in the other study there was no difference between patients with active disease (Disease Activity Score-28-erythrocyte sedimentation rate > 2.6) and those in remission. The same applied to the SLE group. Regardless of the value of the SLE Disease Activity Index, serum adropin levels did not differ significantly [22,25]. This may suggest that in RA and SLE, metabolic disorders and accelerated atherosclerosis have a stronger influence on circulating adropin levels than the inflammatory process itself. Given that RA and SLE are pathophysiologically, very complex diseases mediated by different immune mechanisms, it is almost impossible to balance these two diseases, especially between them and animal models. However, in the absence of human studies, especially in ARDs, the need to interpret results in the context of animal data has become necessary.

In addition to demographic and methodological variability, pharmacological therapy represents an important confounder in interpreting circulating adropin levels in ARDs. Glucocorticoids, widely used in RA and SLE, are known for altering glucose and lipid metabolism, including enhanced hepatic gluconeogenesis, reduced peripheral glucose uptake, hyperglycemia, IR, and dyslipidemia. All of these may indirectly influence *Enho* expression [54,55]. Conventional synthetic disease-modifying antirheumatic drugs such as methotrexate and hydroxychloroquine have been shown to improve metabolic profiles and reduce the risk of type 2 diabetes in RA, while biologics targeting TNF-α and IL-6 signaling may normalize inflammatory–metabolic pathways and even favorably alter body composition [56,57]. Notably, most available studies on adropin in RA, SLE, and other ARDs did not stratify patients according to treatment status, which likely contributes to observed heterogeneity [21,22,24,25]. Future studies should therefore account for therapy type and duration when evaluating adropin as a biomarker in ARDs.

Sex hormones may also regulate circulating adropin. Several studies have shown that women tend to have lower serum adropin levels compared to men, independent of BMI [6,7]. Estrogens and androgens both influence glucose and lipid metabolism, processes in which adropin is deeply involved [58,59]. It is therefore plausible that hormonal differences contribute to the observed variability in adropin levels among ARD patients, since ARDs such as SLE and primary Sjögren’s Syndrome (pSjS), predominantly affect women, while sex differences in RA (2–3:1) and SSc cohorts (4–10:1, up to 8.2:1) could further confound results [60,61]. Stratification by sex and adjustment for hormonal status should be incorporated in future adropin studies.

It has been shown that the expression of *Enho* in liver cells of mouse models is regulated by LXRα and PPAR-γ. Both nuclear factors are highly expressed in RA fibroblast-like synoviocytes and in synovial fluid. Therefore, it can be concluded that the formation of pannus ultimately increases *Enho* expression and affects the levels of adropin in serum [1,25,37,62,63]. Although Gregersen et al. reported genetic variations in *Enho* expression in RA, it remains unclear whether these genetic changes directly affect serum adropin levels in RA, or whether adropin levels are more closely related to disease activity, cardiovascular dysfunction, and disturbances in lipid and glucose metabolism [14,32,40].

Unlike in RA and OA, increased adropin levels were observed in pSjS, SSc, Behçet’s disease (BD), and Kawasaki disease (KD) [21,24,51]. This indicates an association of adropin with chronic inflammation in diseases distinct from RA and OA. First, pSjS and SSc are classified as collagenoses, whereas BD and KD are vasculitides. Although very similar pro-inflammatory cytokines are involved in these disorders, their pathophysiology, organ involvement, and clinical presentation differ significantly [32,64]. Patients with pSjS not only had elevated serum adropin levels compared to healthy controls, but these levels were also positively correlated with high-density lipoprotein and anti-SSA/Ro52 antibodies, and negatively correlated with the Sjögren’s Syndrome Disease Damage Index. Interestingly, no statistically significant correlation was found between adropin levels and EULAR Sjögren’s Syndrome Disease Activity Index. The authors proposed several possible explanations for the observed results [24]. Adropin may modulate NO signaling in pSjS, a condition in which increased activation of eNOS has been demonstrated as a consequence of autoantibodies targeting muscarinic acetylcholine receptors. Consequently, elevated adropin levels may be associated with endothelial protection in pSjS, aiming to reduce atherosclerotic and thrombotic events, which are already more common in affected individuals [29,65,66,67]. The association between adropin and the aforementioned antibodies in pSjS is demonstrated for the first time. Considering previous results, as well as the potentially protective role of SSA/Ro52 in atherogenesis, it can be concluded that higher levels of adropin and SSA/Ro52 positivity, as well as their positive correlation, have a beneficial effect on the atherosclerosis process in pSjS [24,68,69].

Serum adropin levels and *Enho* expression were examined in patients with SSc and BD, compared to healthy controls. As mentioned earlier, statistically significant increases in serum adropin levels was reported in both patient groups compared to healthy controls [21]. The main pathophysiological process of SSc is fibrosis. This process can be influenced by certain adipocytokines, as adiponectin and leptin, which are regulated by adropin [70,71,72,73,74,75,76]. This is a possible connection to increased adropin levels in the SSc cohort. Previous studies have shown elevated leptin levels in SSc patients, while research on animal models has demonstrated that *ob/ob* mice, which have leptin deficiency, exhibit reduced *Enho* expression. Therefore, it could be concluded that in active SSc, where leptin levels are increased, we can consequently expect elevated adropin levels [21,75,77]. The fact that the expression of PPAR-γ, which helps prevent fibrosis, is impaired in patients with SSc undoubtedly indicates the involvement of adropin in the fibrosis process in SSc [1,21,73]. A recently published study showed that therapy with recombinant adropin can inhibit fibrosis. Specifically, exposure of SSc patient skin to recombinant adropin resulted in a decreased number of fibroblasts expressing the transcription factor GLI family zinc finger 1. A reduction in fibroblast activity and number leads to decreased collagen production, thereby limiting fibrosis. It should be emphasized that the results of this study could be clinically applicable [43]. Elevated adropin levels in BD may be explained by the chronic inflammatory milieu, suggesting that inflammation, not just fibrosis, contributes to increased serum adropin levels in SSc [21]. Elevated adropin levels have also been observed in another form of vasculitis—KD. Moreover, KD patients with coronary artery lesions had even higher adropin levels. Based on their results, the authors concluded that adropin could be a measurable biomarker to identify those patients who will develop coronary artery lesions. This study also identified positive correlations between adropin levels and inflammatory markers such as procalcitonin and C-reactive protein. This may reflect a compensatory upregulation of adropin in response to inflammatory stress, supporting its potential protective role in maintaining endothelial integrity [51]. Although the exact pathophysiological mechanisms of adropin’s action in KD have not been fully elucidated, the authors attributed the elevated adropin levels primarily to its effects on the endothelium [78].

Although, the possible pathophysiological roles of adropin in each ARD have been proposed, causal conclusions cannot be drawn due to the lack of sufficiently powered clinical studies. The abovementioned results, as well as discrepancies and key confounders are summarized in Table 1.

## 5. Therapeutic Potential and Translational Perspectives

The increasing prevalence of ARDs in developed countries represents a growing public health concern [79]. Despite numerous studies, ARDs still remain a mystery in terms of pathophysiology, pathogenesis, and treatment. Therefore, there is a continuous need to identify novel biomarkers capable of detecting patients at risk of developing complications at an early stage. Based on current evidence, adropin appears to be a promising candidate.

Adropin is a multifunctional peptide involved in the pathophysiology of numerous conditions, including ARDs [32]. Its most important effects include maintaining metabolic homeostasis, improving glucose and lipid metabolism, and preventing endothelial dysfunction [1,14,80]. Additionally, adropin is involved in various signaling pathways that regulate the balance between pro- and anti-inflammatory states. Reduced levels of adropin can lead to increased activity of pro-inflammatory cytokines, further worsening the course of the ARDs [18]. Although the results of previous studies on adropin and ARDs are not consistent, it is evident that both reduced and elevated serum adropin levels are associated with the pathophysiology of ARDs. ARDs are characterized by accelerated atherosclerosis, increased incidence of cardiovascular diseases, type 2 diabetes, and other comorbidities, which are partly mediated by dysregulation of signaling pathways involving adropin, including NB-3/Notch, AKT/CREB/BDNF, and VEGFR2/PI3K/AKT [12,32,81]. Adropin also affects oxidative stress, macrophage differentiation, and cytokine release [18].

To determine the clinical potential of adropin, its pharmacokinetics must first be defined. As a peptide hormone prone to proteolytic degradation, adropin’s bioavailability may be influenced by the route of administration or therapeutic intervention. It is also necessary to identify the specific population of patients with ARDs in whom it would be appropriate to study adropin. In vitro research should focus on better understanding how these pathways influence the actions of adropin. Ultimately, it is crucial to determine whether the therapeutic effects observed in vitro can be implemented into clinical practice.

The major barrier to the clinical implementation of adropin is the current lack of understanding of the precise mechanisms by which it interferes with the pathophysiology of ARDs. Consequently, there is also a lack of prospective, large-scale studies and robust data from relevant clinical trials. An excellent example of translational potential is provided by a recent study in which recombinant adropin demonstrated promising antifibrotic effects in systemic sclerosis, reducing both the number of fibroblasts and collagen production [43]. These findings support the potential for adropin-based therapies in fibrotic ARDs.

The target population for assessing serum adropin levels in ARDs could include older patients, women, individuals with BMI > 25, and those with dyslipidemia. In these groups, the biology of adropin is further modified. To overcome the limitation of adropin’s short half-life and peptide degradation, the development of adropin analogues or mimetics with improved stability and pharmacokinetics should be considered, which might enable effective systemic administration. The most appropriate route of administration (e.g., subcutaneous, intravenous, or controlled-release formulations) remains to be determined in order to maximize bioavailability and ensure consistent therapeutic effects. In addition, gene therapy approaches or modulation of upstream regulators such as LXRα or PPAR-γ could represent alternative strategies to enhance endogenous adropin expression.

Furthermore, the use of adropin in daily clinical practice could be relatively cost-effective, with good availability and reproducibility once standardized assays and therapeutic formulations are established.

**Figure 2:**
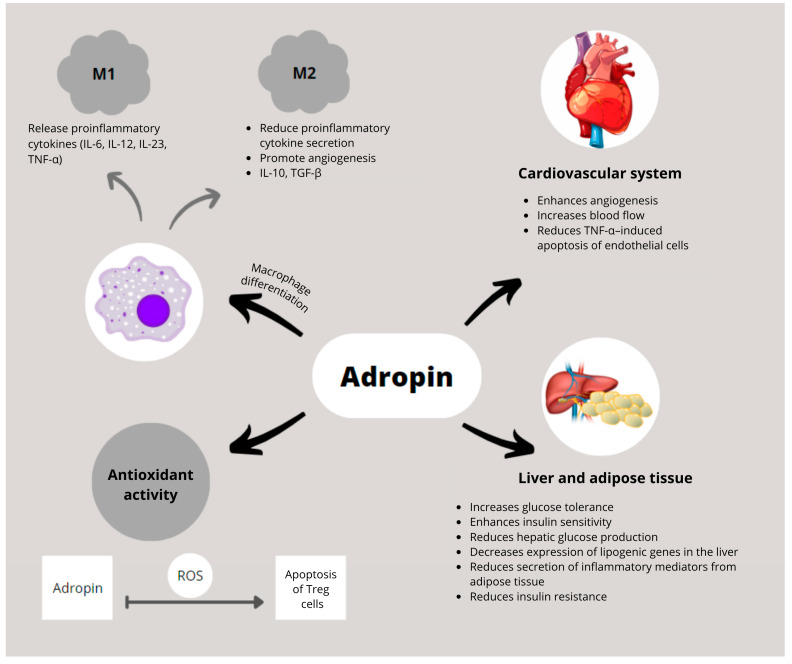
Schematic representation of adropin’s biological functions in immune regulation, cardiovascular protection, and metabolic homeostasis. Adropin promotes polarization of macrophages toward the anti-inflammatory M2 phenotype and suppresses pro-inflammatory cytokine production by M1 macrophages. In the cardiovascular system, adropin enhances angiogenesis, improves blood flow, and inhibits TNF-α-induced endothelial cell apoptosis. In liver and adipose tissue, it improves glucose tolerance and insulin sensitivity, reduces hepatic glucose production and lipogenic gene expression, and decreases secretion of inflammatory mediators. Adropin also exerts antioxidant effects by limiting ROS-induced apoptosis of regulatory Tregs. Abbreviations: IL-10: interleukin 10; IL-6: interleukin 6; IL-12: interleukin 12; IL-23: interleukin 23; ROS: reactive oxygen species; TGF-β: transforming growth factor beta; TNF-α: tumor necrosis factor alpha; Treg: regulatory T cell.

**Figure 3:**
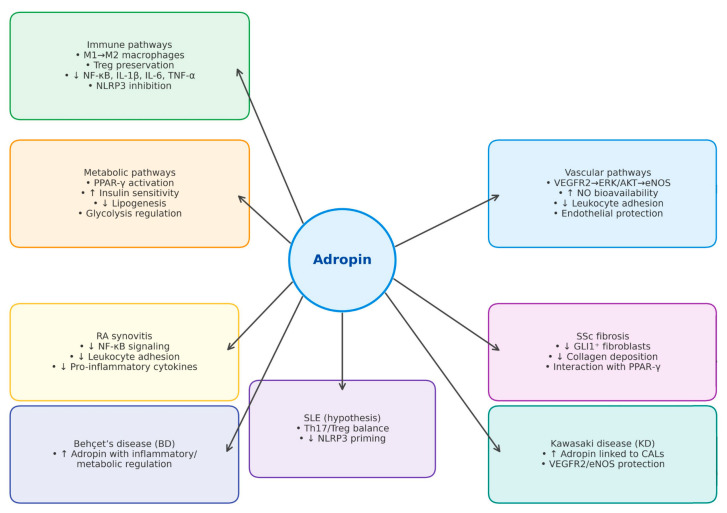
Disease-specific pathways in autoimmune rheumatic diseases (ARDs). The schematic highlights key molecular and cellular mechanisms implicated in the pathogenesis of rheumatoid arthritis (RA), systemic lupus erythematosus (SLE), systemic sclerosis (SSc), Behçet’s disease (BD), primary Sjögren’s syndrome (pSjS), and Kawasaki disease (KD). Upward arrows (↑) indicate upregulation or increase, while downward arrows (↓) indicate downregulation or decrease. Abbreviations: AKT: protein kinase B; BD: Behçet’s disease; ERK1/2: extracellular signal-regulated kinase ½; ECs: endothelial cells; IL: interleukin; KD: Kawasaki disease; MPO-ANCA: myeloperoxidase anti-neutrophil cytoplasmic antibodies; NF-κB: nuclear factor kappa B; NO: nitric oxide; PPAR-γ: peroxisome proliferator activated receptor-γ; RA: rheumatoid arthritis; SLE: systemic lupus erythematosus; SSc: systemic sclerosis; TNF-α: tumor necrosis factor α; Tregs: regulatory T cells; VEGFR2: vascular endothelial growth factor receptor 2; eNOS: endothelial nitric oxide synthase; pSjS—primary Sjögren’s syndrome; NLRP3: NOD-like receptor family pyrin domain-containing 3; CALs: coronary artery lesions; GLI1: glio-ma-associated oncogene homolog 1; Th17: T helper 17 cells.

The biological functions of adropin in immune regulation, cardiovascular protection, and metabolic homeostasis are summarized in Figure 2. Figure 3 illustrates disease-specific pathways in ARDs.

The main limitations of this review reflect those of the available literature: small sample sizes, methodological differences, and lack of stratification for critical confounders, all of which increase between-study heterogeneity. Future studies should therefore be standardized with respect to sample size per disease, patient age and sex, disease duration and activity, BMI/adiposity and IR, cytokine milieu, pharmacological therapy (glucocorticoids, conventional synthetic and targeted synthetic disease-modifying antirheumatic drugs, biologics), sex hormones, endothelial and fibrotic involvement, as well as preanalytical variables such as fasting status. It is essential to perform multivariable adjustment for these confounders. Only with that homogeneity will it be possible to draw more concrete conclusions about the clinical usefulness of adropin in everyday practice.

## 6. Conclusions

In conclusion, current evidence indicates that further research is needed to clarify the pathophysiology of ARDs and to identify novel measurable biomarkers that could predict the risk of developing comorbidities. Although there is still a long way to go from the current knowledge of adropin to its eventual implementation in clinical practice, we believe that further well-designed studies will provide an answer to this question.

## Figures and Tables

**Table 1:** Summary of studies on circulating adropin in autoimmune rheumatic diseases (ARDs), with main findings and key confounders.

Disease	References	Main Findings	Key Confounders
Rheumatoid Arthritis (RA)	[22,25]	Decreased serum adropin compared to controls; inverse correlation with TNF-α and glucose metabolism; inconsistent *Enho* expression findings	BMI and IR; disease duration; age; treatment status
Systemic Lupus Erythematosus (SLE)	[22]	No significant change in serum adropin compared to controls	Small sample size; heterogeneous cohorts; treatment effects not stratified
Osteoarthritis (OA)	[23]	Lower adropin correlated with greater OA severity, TNF-α, WBC, and NLR; lowest levels in patients with BMI ≥ 30	Obesity; inflammatory burden; comorbid metabolic syndrome
Psoriasis (with MetS)	[50]	Decreased adropin in psoriasis; lowest in patients with MetS; associated with metabolic alterations	BMI; presence of metabolic syndrome
Primary Sjögren’s Syndrome (pSjS)	[24]	Increased adropin; positively correlated with HDL and anti-SSA/Ro52; negatively with SSDDI	Female predominance; autoantibody status; endothelial risk factors
Systemic Sclerosis (SSc)	[21,43]	Increased adropin; associated with fibrosis modulation via GLI1 and PPAR-γ; potential antifibrotic role	Leptin and adipokine imbalance; impaired PPAR-γ; disease duration; therapy
Behçet’s Disease (BD)	[21]	Increased adropin; potentially linked to inflammatory/metabolic dysregulation	Inflammatory burden; vascular involvement
Kawasaki Disease (KD)	[51]	Increased adropin in CAL + KD patients; correlated with CRP and procalcitonin	Pediatric cohort; acute inflammation; coronary artery lesions

Abbreviations: *Enho*: energy homeostasis-associated gene; TNF-α: tumor necrosis factor alpha; IR: insulin resistance; WBC: white blood cell count; NLR: neutrophil-to-lymphocyte ratio; BMI: body mass index; MetS: metabolic syndrome; HDL: high-density lipoprotein; SSDDI: Sjögren’s Syndrome Disease Damage Index; GLI1: GLI family zinc finger 1; PPAR-γ: peroxisome proliferator-activated receptor gamma; CAL: coronary artery lesions; CRP: C-reactive protein.

## Data Availability

Not applicable.

## Abbreviations

The following abbreviations are used in this manuscript:

AKT	protein kinase B
ARDs	autoimmune rheumatic diseases
BD	Behçet’s disease
BMI	body mass index
ERK1/2	extracellular signal-regulated kinase 1/2
ECs	endothelial cells
*Enho*	energy homeostasis-associated
IL	interleukin
IR	insulin resistance
KD	Kawasaki disease
LXRα	liver X receptor alpha
MPO-ANCA	myeloperoxidase anti-neutrophil cytoplasmic antibodies
NF-κB	nuclear factor kappa B
NO	nitric oxide
OA	osteoarthritis
PPAR-γ	peroxisome proliferator activated receptor-γ
RA	rheumatoid arthritis
SLE	systemic lupus erythematosus
SSc	systemic sclerosis
TNF-α	tumor necrosis factor α
Tregs	regulatory T cells
VEGFR2	vascular endothelial growth factor receptor 2
eNOS	endothelial nitric oxide synthase
mRNA	messenger ribonucleic acid
pSjS	primary Sjögren’s Syndrome
